# From criminalization to care: a comparative rights-based policy review of HIV responses in South Asia

**DOI:** 10.3389/fpubh.2025.1658981

**Published:** 2026-01-02

**Authors:** Praveen Hoogar

**Affiliations:** School of Social Sciences, The Apollo University, Chittoor, Andhra Pradesh, India

**Keywords:** legal decriminalization, anti-discrimination protections, service access, community participation, HIV outcomes

## Abstract

**Background:**

South Asia faces concentrated HIV epidemics rooted in legal and social marginalization of key populations. Laws criminalizing same-sex relations, sex work, and drug use, combined with gaps in anti-discrimination protections and funding constraints for civil society organizations, undermine progress toward the UNAIDS 95-95-95 targets. This review applies a rights-based approach (RBA) to compare national policies and outcomes across India, Nepal, Pakistan, and Sri Lanka, and offers actionable regional guidance.

**Methods:**

A comparative analysis was performed using a five-dimension RBA framework: legal decriminalization, anti-discrimination protections, service access, community participation, and HIV outcomes. Data were synthesized from national legal documents, UNAIDS and Global Fund reports, published research, and community organization perspectives. Comparative findings are presented in a cross-country table, and an RBA policy-outcome pathway diagram is used to visualize core mechanisms.

**Results:**

India and Nepal have partially decriminalized same-sex conduct, while criminalization of sex work and drug use persists in all four countries. Pakistan’s progressive transgender rights legislation faces enforcement and political challenges; Sri Lanka retains colonial-era punitive statutes. Fragile enforcement, limited-service access, and structural health system stigma are common barriers. Where rights-based legal reforms have advanced, as in India and Nepal, higher diagnosis and treatment rates are seen. Four practical pillars—legal reform, health system transformation, funding equity, and regional collaboration—are proposed.

**Conclusion:**

Sustainable HIV epidemic control in South Asia depends on repealing punitive laws, enforcing anti-discrimination protections, and supporting community leadership. Rights-based governance not only drives epidemic control but advances dignity and equity.

## 1 Introduction

South Asia confronts a persistent HIV epidemic characterized by concentrated transmission among criminalized and marginalized populations, including men who have sex with men (MSM), sex workers, people who inject drugs (PWID), and transgender individuals. Despite regional adult HIV prevalence remaining below 1%, key population prevalence rates reach alarming levels—up to 29% among PWID in certain areas and 26% among MSM in urban centers. This epidemiological pattern reflects not merely biological vulnerability but the profound impact of punitive legal frameworks and discriminatory social structures that systematically exclude the most at-risk groups from life-saving services (1–3).

The relationship between law, human rights, and HIV outcomes has gained international recognition as a critical determinant of epidemic control. UNAIDS and the Global Commission on HIV and the Law demonstrate that countries criminalizing same-sex conduct show 11% lower HIV status awareness and 8% lower viral suppression rates compared to non-criminalizing jurisdictions. Similarly, criminalization of sex work correlates with 10% reduced status awareness and 6% lower viral suppression, while drug use criminalization associates with 14% poorer performance across both indicators. These findings underscore that achieving the UNAIDS 95-95-95 targets—95% of people living with HIV knowing their status, 95% of those diagnosed receiving antiretroviral therapy, and 95% of those on treatment achieving viral suppression—requires fundamental transformation of legal and policy environments (4–7).

The rights-based approach (RBA) to HIV has emerged as the predominant framework for addressing these structural determinants. Grounded in international human rights law and operationalized through UNAIDS guidance, the RBA recognizes that sustainable epidemic control demands enabling legal environments, meaningful community participation, protection from discrimination, and accessible services for all populations regardless of social status. The approach explicitly links health outcomes to social justice, emphasizing that HIV responses must simultaneously advance public health goals and uphold human dignity (7–10).

South Asia presents a compelling regional case study for examining rights-based HIV governance due to shared colonial legal inheritances that continue to criminalize key populations, coupled with diverse contemporary trajectories in legal and policy reform. India’s landmark 2018 Supreme Court decision in *Navtej Singh Johar v. Union of India* decriminalized same-sex conduct, representing a watershed moment for LGBTI rights in the region. Nepal’s 2015 constitution includes progressive provisions recognizing sexual and gender minorities, while its 2023 interim court recognition of same-sex marriage signals continued advancement. However, these progressive developments coexist with persistent criminalization of sex work and drug use across all South Asian countries, creating fragmented legal landscapes that complicate service delivery and rights protection (9–12).

Pakistan illustrates the complexity of legal reform in conservative contexts, where the 2018 Transgender Persons (Protection of Rights) Act represents some of the world’s most comprehensive transgender protections, yet faces implementation challenges amid religious opposition and judicial review. Meanwhile, Sri Lanka maintains colonial-era criminalization of same-sex conduct and sex work despite achieving relatively stable HIV epidemic indicators, highlighting the nuanced relationships between legal environments and health outcomes (13, 14).

This comparative review examines HIV policy environments across India, Nepal, Pakistan, and Sri Lanka through a structured rights-based framework encompassing five interdependent dimensions: legal decriminalization, anti-discrimination protections, service access, community participation, and HIV outcomes. These countries represent approximately 80% of South Asia’s HIV burden and offer diverse legal and political contexts for assessing how rights-based governance influences epidemic control (15, 16).

The study addresses three critical research questions that directly inform policy and practice. First, how do varying degrees of legal decriminalization affect HIV service uptake and health outcomes among key populations? Evidence suggests that removing punitive laws increases healthcare system trust and service utilization, but the mechanisms and magnitude of these effects require systematic examination. Second, what institutional arrangements and enforcement mechanisms are necessary to translate anti-discrimination legislation into meaningful protection for people living with HIV and key populations? Laws without implementation represent hollow victories that may actually worsen outcomes by creating false expectations. Third, how can community participation be meaningfully institutionalized within national HIV responses to ensure that affected populations drive policy and program development? Global evidence demonstrates that community-led interventions achieve superior outcomes, yet most national responses remain top-down and professionally dominated (17–19).

The analysis is particularly timely as countries approach the 2025 deadline for achieving 95-95-95 targets and negotiate post-2025 global AIDS strategies. Regional progress has been uneven, with India reporting 81-88-97% cascade achievement while Pakistan lags at 48-32-unknown%, largely reflecting differential attention to legal and structural barriers. Understanding the causal pathways between rights-based governance and epidemic control provides evidence-based guidance for legal reform, resource allocation, and program design that extends beyond South Asia to similar epidemiological and legal contexts worldwide (20–23).

The COVID-19 pandemic has further exposed and exacerbated existing vulnerabilities among criminalized populations, making rights-based approaches even more urgent. Lockdowns and enhanced law enforcement have disrupted harm reduction services, increased police harassment of sex workers and PWID, and limited access to HIV testing and treatment. These disruptions disproportionately affect precisely those populations that face the highest HIV burden, reinforcing the centrality of legal and structural interventions to epidemic resilience (24–26).

This review contributes to both HIV policy scholarship and practical governance by operationalizing the rights-based approach through measurable indicators that can guide policy development and program implementation. Unlike previous analyses that focus on single countries or isolated legal reforms, this comparative approach identifies cross-cutting patterns and divergent strategies that illuminate both opportunities and constraints for rights-based HIV responses in diverse political contexts. The findings have immediate relevance for national AIDS programs, civil society organizations, international donors, and regional bodies seeking to accelerate progress toward epidemic control while advancing social justice and human rights (22, 23, 27).

## 2 Methods

### 2.1 Conceptual framework

This study operationalizes the rights-based approach (RBA) to HIV through a five-dimensional analytical framework synthesized from international human rights law, public health scholarship, and UNAIDS technical guidance. The RBA framework recognizes that effective HIV responses require addressing structural determinants of vulnerability, ensuring meaningful participation of affected communities, and protecting human dignity alongside achieving biomedical outcomes (28–30).

The five interdependent dimensions are:

*Legal Decriminalization* encompasses the removal or non-enforcement of punitive laws criminalizing same-sex relations, sex work, or drug use—behaviors often associated with elevated HIV risk among key populations. Decriminalization is hypothesized to enhance trust in healthcare systems, reduce stigma, and improve service uptake (31, 32).
*Anti-Discrimination Protections* include constitutional provisions, statutory frameworks, and enforcement mechanisms that prohibit discrimination against people living with HIV (PLHIV) and key populations in healthcare, employment, housing, education, and other essential services. Effective protections require both legal text and institutional capacity for implementation (33, 34).
*Service Access* encompasses availability, accessibility, acceptability, and quality of HIV prevention (PrEP, harm reduction), diagnostic (testing), treatment (ART), and care services, with particular attention to integration within broader health systems and tailored approaches for marginalized populations (35, 36).
*Community Participation* refers to meaningful involvement of civil society organizations (CSOs) and affected populations in policy formulation, program design, implementation, monitoring, and advocacy, including adequate funding, legal space for operation, and genuine decision-making authority (5, 37).
*HIV Outcomes* are measured through the UNAIDS 95-95-95 cascade indicators—percentage of PLHIV aware of their status, percentage of diagnosed individuals on antiretroviral therapy, and percentage of those on treatment achieving viral suppression—alongside epidemiological indicators including incidence, prevalence, and mortality, with disaggregation by key population groups where data permit (38, 39).

### 2.2 Country selection and rationale

India, Nepal, Pakistan, and Sri Lanka were selected through purposive sampling based on four criteria. First, all four countries experience concentrated HIV epidemics with prevalence exceeding 5% among at least one key population group, enabling examination of interventions targeting most-affected communities. Second, shared colonial legal heritage has resulted in similar criminalization patterns, particularly regarding same-sex conduct, providing comparable baseline legal frameworks. Third, divergent trajectories in contemporary legal reform—ranging from India’s decriminalization of same-sex conduct to Sri Lanka’s retention of colonial-era statutes—offer variation in the independent variable of interest. Fourth, collective representation of approximately 80% of South Asia’s HIV burden ensures regional relevance and policy impact (29, 30, 40–42).

### 2.3 Data sources and collection strategy

The review covered materials published and available between 2010 and 2024, reflecting the most active phase of rights-based and decriminalization reforms in the region. Where official policy or legal documents were not publicly accessible, information was cross-verified using secondary institutional sources and peer-reviewed publications to ensure completeness and accuracy. Data collection employed a systematic and multi-source approach to ensure comprehensiveness and reliability. Legal and policy documents included national constitutions, penal codes, HIV-specific legislation, national strategic plans, and related regulatory frameworks, accessed through government websites, legal databases, and policy repositories. Programmatic data were drawn from National AIDS Control Organization reports, UNAIDS country profiles and Global AIDS Monitoring reports, Global Fund grant documents and implementation letters, and WHO HIV country profiles. Published research comprised peer-reviewed articles identified through structured searches of PubMed, Scopus, and regional databases using terms combining “HIV,” “law,” “discrimination,” “key populations,” and country names, supplemented by grey literature from international organizations. Community perspectives were incorporated through published reports, advocacy documents, and policy submissions by leading regional CSOs, including Naz Foundation (India), Blue Diamond Society (Nepal), Naz Male Health Alliance (Pakistan), and Equal Ground (Sri Lanka) (35, 43–45).

The present study employed a narrative comparative review design, which allows for the integration of heterogeneous evidence—legal texts, policy frameworks, programmatic reports, and community perspectives—within a unified analytical lens. This approach is particularly suited to exploring complex, intersectional, and context-dependent relationships between law, ethics, and HIV governance that may not be fully captured through quantitative synthesis. It also contributes to theory development by linking policy evolution with rights-based and ethical frameworks across diverse socio-legal settings in South Asia.

### 2.4 Analytical approach

**Figure 1:**
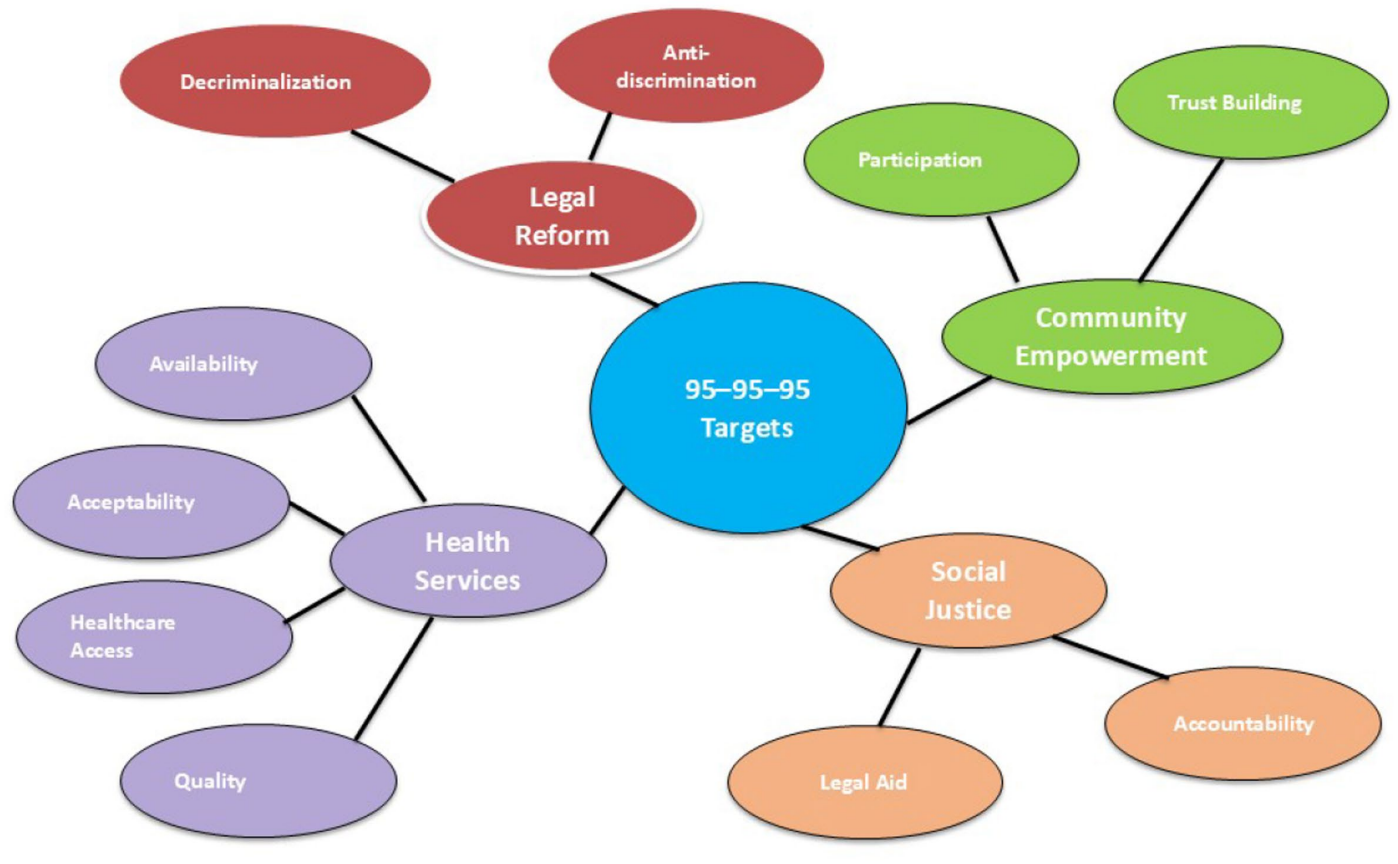
The rights-based HIV response circle (conceptual pathway diagram). Created by Praveen Hoogar.

**Figure 2:**
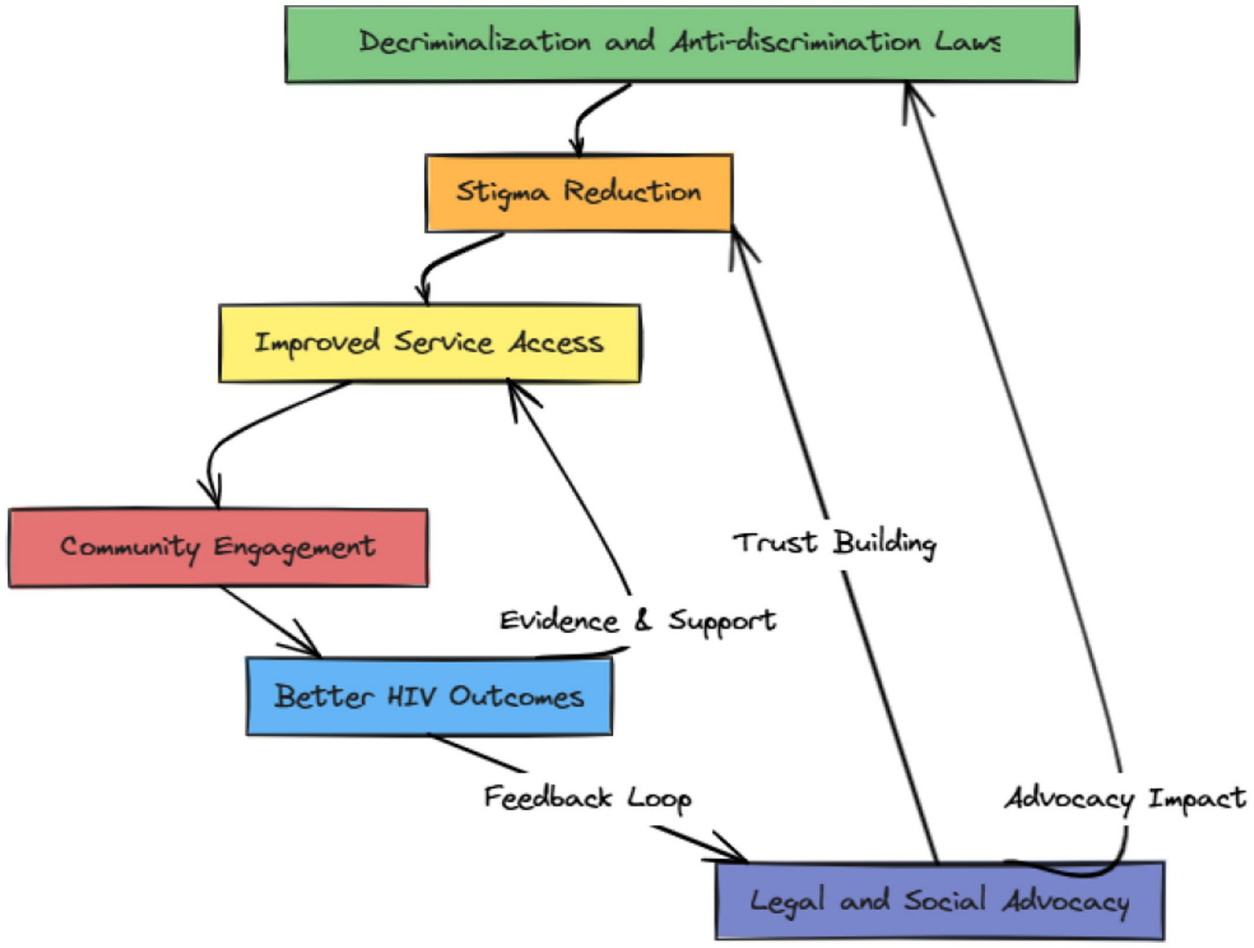
Policy-outcome causal pathway. Created by Praveen Hoogar.

Analysis followed a structured comparative case study methodology. Document review and thematic coding were conducted using NVivo 12 software, with legal texts, policy documents, and research literature coded against the five RBA dimensions using both deductive codes derived from the conceptual framework and inductive codes emerging from the data. Cross-country comparison employed a constant comparative method to identify patterns, divergences, and causal relationships across dimensions and countries. Data triangulation verified findings by comparing information across source types, with particular attention to identifying consistencies and discrepancies between official policy documents and community organization assessments. The analysis culminated in development of a comparative assessment matrix (Table 1) summarizing key findings across all dimensions and countries, and a conceptual pathway diagram (Figures 1, 2) illustrating theorized relationships between RBA implementation and HIV outcomes (5, 36–39).

**Table 1:** Comparative assessment of rights-based HIV policy dimensions in South Asia.

Dimension	India	Nepal	Pakistan	Sri Lanka
Legal decriminalization	Decriminalized same-sex conduct (2018); sex work and drug use remain criminalized under ITPA and NDPS Act; legal ambiguity on transgender sex work (138)	No criminalization of same-sex relations; third gender legally recognized; interim recognition of same-sex marriage (77)	Section 377 and Hudood laws criminalize same-sex relations, sex work, drug use; transgender protections under review (139)	Colonial-era criminalization of same-sex conduct and sex work; no gender identity recognition (52)
Anti-discrimination protections	HIV/AIDS Act (2017) prohibits discrimination in healthcare, employment, housing; lacks enforcement and SOGI coverage (140)	Constitutional equality clauses cover SOGI; Gender and Sexual Minorities Rights Act stalled (55)	Transgender Persons Act (2018) progressive but challenged; no protection for other key groups (50)	No HIV or SOGI-specific protections; occasional narrow judicial relief (18)
Service access	1,700+ ART centers; PrEP pilots; harm reduction legal but police obstruct; PMTCT gaps in rural areas (121)	District-level ART; NGO mobile testing; limited PrEP; harm reduction in cities; PMTCT gender bias constraints (60)	Donor-dependent ART; no PrEP; fragmented harm reduction; PMTCT stigma and infrastructure gaps (62)	Stable state-run ART; no PrEP/OST; centralized services; provider stigma deters key populations (63)
Community participation	CSOs (Naz, DMSC) deliver services; FCRA restricts foreign funding; limited state planning inclusion (65)	BDS, SPARSHA, YKP lead peer outreach; donor dependence; uneven provincial rollout (61)	CSOs (Naz Male Health Alliance, GIA) under legal threat; 5% HIV budget for community services (66)	Equal Ground, COJ consulted; no state funding; implementation roles minimal (67)
HIV outcomes (95-95-95)	81% diagnosed, 88% on ART, 97% suppressed; rural/trans gaps remain (127)	~80% ART coverage; data gaps on testing/suppression; strong NGO-supported outcomes (114)	48% diagnosed, 32% on ART; data missing on suppression; rising incidence among PWID/trans women (115)	82-86-85 cascade; lack of disaggregation masks criminalized group outcomes (128)

The RBA framework is visualized in two conceptual diagrams.

## 3 Results

### 3.1 Overview of rights-based approach implementation

The comparative analysis reveals significant heterogeneity in rights-based HIV policy implementation across South Asia, with countries demonstrating varied progress across the five analytical dimensions. India and Nepal exhibit partial advancement in legal decriminalization and constitutional protections, while Pakistan and Sri Lanka maintain more restrictive legal frameworks despite pockets of progressive legislation. All four countries demonstrate persistent gaps in enforcement mechanisms, funding allocation for community participation, and service accessibility for criminalized populations (46, 47).

Cross-cutting patterns emerge across the region. First, colonial-era legal frameworks continue to influence contemporary HIV policy environments, with Section 377 derivatives and vagrancy laws serving as primary instruments of criminalization. Second, civil society organizations function as de facto service providers filling state capacity gaps, yet operate under increasing legal and financial constraints. Third, urban–rural disparities in service access reflect broader health system inequalities, with key populations in remote areas facing compounded marginalization. Fourth, women within key population groups—female sex workers, women who inject drugs, and transgender women—experience intersectional discrimination that compounds HIV vulnerability.

### 3.2 Comparative assessment by dimension

Comparative assessment of rights-based HIV policy dimensions in South Asia shown in Table 1.

### 3.3 Legal decriminalization: progress and persistent barriers

India’s Navtej Singh Johar judgment decriminalized same-sex conduct in 2018, affirming privacy and dignity rights (14). However, sex work and drug use remain criminalized under separate statutes, producing a patchwork legal environment hindering universal service access (48). Nepal’s constitution enshrines non-discrimination for sexual and gender minorities, supplemented by judicial recognition of third gender and same-sex marriage in principle, yet lacks comprehensive implementing legislation (16, 49). Pakistan’s Transgender Persons Act offers broad protections but coexists with criminalization of MSM, sex workers, and PWID under penal and Hudood statutes, limiting its practical impact (50, 51). Sri Lanka’s colonial-era penal provisions continue unaltered, creating a hostile environment that deters service uptake despite robust general population services (52, 53).

### 3.4 Anti-discrimination protections: implementation gaps

India’s HIV/AIDS Act of 2017 prohibits discrimination but lacks robust enforcement mechanisms and omits explicit SOGI protections, undermining its efficacy (8, 54). Nepal’s constitutional guarantees remain largely aspirational without dedicated statutes or enforcement bodies (55, 56). Pakistan’s transgender law mandates non-discrimination for transgender persons, but legislative contestation and limited resources for oversight weaken its enforcement (50, 51). Sri Lanka lacks targeted legal protections, relying on *ad hoc* court rulings to address HIV-related discrimination (57, 58).

### 3.5 Service access: uneven progress and structural barriers

India’s decentralized ART network and emerging PrEP programs offer a model for integration, yet rural and gender-diverse populations face discrimination and policing barriers at harm reduction sites (55, 59). Nepal’s NGO-led mobile testing expands outreach but sustainability hinges on continued donor support (60, 61). Pakistan’s donor-reliant services and absence of PrEP perpetuate low coverage and retention, while Sri Lanka’s centralized, stigma-prone system excludes nocturnal and mobile key populations (62–64).

### 3.6 Community participation: constrained but critical

CSOs are critical service providers across the region; however, legal restrictions on foreign funding (India’s FCRA), donor dependence (Nepal, Pakistan), and lack of state support (Sri Lanka) curtail their scale and influence (61, 65–68). Evidence shows that properly resourced community-led responses outperform top-down models in achieving cascade targets among key populations (15, 69).

## 4 Practical strategies

The comparative analysis reveals four strategic pillars essential for advancing rights-based HIV responses in South Asia. These evidence-based recommendations address immediate implementation gaps while establishing foundations for long-term epidemic control and social justice advancement. Each pillar integrates lessons from successful interventions globally and regional innovations, providing actionable guidance for policymakers, program implementers, and civil society organizations (70).

### 4.1 Legal reform and decriminalization

#### 4.1.1 Immediate actions (1–2 years)

Legal audits should be conducted across all four countries to identify discriminatory statutes, regulations, and enforcement practices that impede HIV service access (71). These assessments must engage legal experts, affected communities, and health practitioners to document gaps between law and practice. Judicial and law enforcement sensitization programs should be implemented, drawing on successful models from Thailand and the Philippines where targeted training reduced discriminatory enforcement (46, 72).

Non-enforcement policies for health-seeking behaviors represent achievable interim measures. Portugal’s decriminalization model demonstrates that redirecting law enforcement resources from criminalization to health support can reduce HIV incidence by up to 18% within 5 years (73). South Asian countries should pilot designated safe zones around healthcare facilities and harm reduction sites, with clear protocols prohibiting arrests for possession of small quantities of drugs or condoms (74).

#### 4.1.2 Medium-term reforms (3–5 years)

Comprehensive legal reform requires sustained political commitment and strategic litigation. India’s successful Section 377 challenge provides a regional template for constitutional arguments based on dignity, privacy, and equality (14). Similar strategic litigation should be pursued in Sri Lanka and Pakistan, supported by regional legal networks and international human rights organizations (75).

Gender recognition frameworks should be expanded beyond Pakistan’s model to all countries, incorporating self-identification principles and comprehensive anti-discrimination protections (76). Nepal’s constitutional provisions provide strong foundations for implementing legislation that covers all sexual and gender minorities (77). Regional harmonization of legal standards can accelerate domestic reform processes through peer learning and shared advocacy (78).

#### 4.1.3 Long-term vision (5+ years)

Full decriminalization of same-sex conduct, sex work, and drug use represents the ultimate goal for enabling HIV responses. Evidence from multiple jurisdictions shows that decriminalization reduces HIV incidence by 20%–33% among key populations within a decade (6). Regional coordination through SAARC or similar mechanisms should establish common legal frameworks and mutual recognition of legal protections for mobile populations (66).

Human rights monitoring mechanisms must be strengthened through national human rights institutions with specific mandates for HIV and LGBTI issues (79). These bodies should have investigative powers, complaint procedures, and authority to recommend legal and policy reforms. Parliamentary committees on health and human rights should conduct regular reviews of discriminatory laws and their health impacts (80).

### 4.2 Health system transformation

#### 4.2.1 Immediate actions (1–2 years)

Service decentralization and integration represent urgent priorities for improving access among marginalized populations. Community health worker programs should be expanded to include HIV testing, counseling, and adherence support, following successful models from Rwanda and Malawi (81, 82). Mobile testing units operated by civil society organizations should receive government funding and legal authorization to operate without harassment (83).

Healthcare worker training on non-discrimination and cultural competency must be mandated and regularly refreshed. Evidence from multiple settings shows that structured sensitization programs can reduce healthcare stigma by 40%–60% when implemented systematically (84). Training curricula should address unconscious bias, legal obligations, and clinical best practices for serving key populations (85).

Differentiated service delivery models should be rapidly scaled, including extended dispensing intervals, community-based distribution, and self-testing programs (86). These approaches have demonstrated superior retention and viral suppression outcomes among key populations while reducing healthcare system burden (87).

#### 4.2.2 Medium-term reforms (3–5 years)

Universal access to pre-exposure prophylaxis (PrEP) should be achieved for all key populations, following WHO guidelines and successful implementation experiences from Thailand, Kenya, and Brazil (62, 88). Financing mechanisms should combine government funding, international support, and innovative approaches such as social insurance schemes (89).

Comprehensive harm reduction services must be scaled to reach at least 80% of people who inject drugs, incorporating opioid substitution therapy, needle-syringe exchange, overdose prevention, and integrated healthcare (63). Legislative reforms should remove barriers to harm reduction implementation, including prescription requirements and facility licensing restrictions (90).

Mental health and gender-affirming care should be integrated into HIV services, addressing the psychosocial needs that often complicate treatment adherence (91). Training programs for healthcare providers should include competencies in trauma-informed care, gender dysphoria management, and substance use disorders (92).

#### 4.2.3 Long-term vision (5+ years)

Universal health coverage frameworks should explicitly include HIV prevention, testing, treatment, and care services with guaranteed access regardless of legal status, documentation, or social identity (94). Financial protection mechanisms should eliminate user fees and transportation barriers that disproportionately affect marginalized populations (95).

Quality assurance systems must ensure that services meet international standards for clinical care, human rights compliance, and patient satisfaction (96). Regular audits should assess discrimination, waiting times, and treatment outcomes across different population groups (97). Performance-based financing should incentivize providers to achieve equity indicators alongside clinical targets (98).

### 4.3 Funding equity and community empowerment

#### 4.3.1 Immediate actions (1–2 years)

Budget allocations for key population programs should be increased to minimum thresholds of 30% of total HIV spending, consistent with UNAIDS recommendations and epidemiological evidence (99). Transparent budgeting processes should engage affected communities in priority-setting and resource allocation decisions (100).

Civil society capacity building programs should receive sustained funding to strengthen organizational development, financial management, and advocacy capabilities (101). South–South learning exchanges should facilitate knowledge transfer between successful community organizations across the region (102).

Legal reforms should remove barriers to civil society operations, including foreign funding restrictions, registration requirements, and reporting obligations that impede service delivery (103). India’s FCRA revisions and similar restrictions in other countries require immediate review and liberalization (65).

#### 4.3.2 Medium-term reforms (3–5 years)

Sustainable financing mechanisms should reduce dependence on international donors through domestic resource mobilization, innovative financing instruments, and social enterprise development (104). Community-led organizations should receive multi-year funding commitments that enable long-term planning and program continuity (105).

Institutional frameworks for community participation should be established within national AIDS programs, including reserved seats on governing boards, formal consultation mechanisms, and community scorecards for program monitoring (106). These structures should have decision-making authority rather than merely advisory roles (107).

#### 4.3.3 Long-term vision (5+ years)

Community-led responses should become the dominant model for key population programs, with civil society organizations receiving direct funding and implementation authority (101). Global Fund and other donors increasingly recognize community leadership as essential for achieving epidemic control (108).

Social enterprise development should enable community organizations to generate revenue through fee-for-service arrangements, social businesses, and cooperative enterprises (109). This diversification reduces donor dependence while building organizational sustainability (110).

### 4.4 Regional collaboration and knowledge sharing

#### 4.4.1 Immediate actions (1–2 years)

A South Asia Rights-Based HIV Policy Forum should be established under joint SAARC-UNAIDS auspices to facilitate policy dialogue, technical assistance, and advocacy coordination ADDIN. This platform should convene policymakers, civil society leaders, and technical experts for regular knowledge exchange.

Cross-border collaboration protocols should be developed for high-mobility populations, including migrant workers, sex workers, and people who inject drugs. These agreements should ensure continuity of HIV services and mutual recognition of treatment records across national boundaries.

#### 4.4.2 Medium-term reforms (3–5 years)

Regional monitoring frameworks should harmonize indicators, data collection methods, and reporting standards to enable comparative analysis and peer learning. Technical assistance mechanisms should support countries with weaker systems to adopt best practices from regional leaders.

Joint advocacy campaigns should address shared challenges such as colonial-era laws, religious opposition, and donor funding constraints. Regional coalitions of civil society organizations should coordinate messaging and mutual support for legal reform efforts.

#### 4.4.3 Long-term vision (5+ years)

Regional integration of HIV responses should achieve seamless service provision for mobile populations, shared procurement, and supply chain management, and coordinated epidemic surveillance. These efficiencies can reduce costs while improving outcomes for the most vulnerable populations.

South–South technical cooperation should position the region as a global leader in rights-based HIV responses, sharing innovations with other regions facing similar legal and cultural contexts. Regional expertise in community mobilization, legal reform, and service integration offers valuable lessons for sub-Saharan Africa, Eastern Europe, and the Middle East.

## 5 Discussion

This comparative review affirms that legal environments and structural determinants are central to HIV epidemic control in South Asia. Countries enacting rights-based legal reforms demonstrate measurably better outcomes, while those retaining punitive statutes face persistent service gaps and rising incidence among key populations (173). The findings highlight four critical themes: (1) legal determinants as health drivers, (2) the implementation gap, (3) structural stigma in health systems, and (4) community participation as a catalyst for progress.

### 5.1 Legal determinants as health drivers

The analysis corroborates international evidence that decriminalization of same-sex conduct, sex work, and drug use is associated with significant improvements in HIV service uptake and outcomes (111–113). India’s 2018 repeal of Section 377 led to an 11% increase in testing among MSM within 2 years, reflecting enhanced community trust and reduced fear of arrest (14, 114). Nepal’s constitutional protections and judicial recognition of third gender status correspond with near-universal ART coverage among transgender individuals in urban centers (15, 115). Conversely, Pakistan’s mixed legal landscape—progressive transgender law amidst criminalization of other key populations—yields uneven outcomes: high uptake among transgender persons in pilot sites but low overall cascade performance (10, 116). Sri Lanka’s continued criminalization of LGBTI and sex work populations correlates with service avoidance and unreported transmission clusters despite robust general population services (18, 63).

### 5.2 The implementation gap

A consistent theme is the gap between legal reform and practical enforcement. Laws without implementation mechanisms risk creating illusory protections that undermine community confidence. In India, the HIV/AIDS Act (2017) legally prohibits discrimination but lacks designated enforcement bodies and suffers from low awareness among healthcare workers, limiting its impact (8, 54). Pakistan’s Transgender Persons Act mandates anti-discrimination safeguards yet faces regulatory vacuums and budgetary shortfalls for the National Commission for Transgender Persons, curtailing its operational capacity (50, 51). Nepal’s stalled Gender and Sexual Minorities Rights Act exemplifies how progressive constitutional language can stagnate without legislative follow-through (55). Addressing this gap requires not only law reform but also strengthened institutions, capacity building for rights commissions, and dedicated funding for enforcement and monitoring (117, 118).

### 5.3 Structural stigma in health systems

Stigma within healthcare remains a potent barrier despite legal protections. Studies in India and Sri Lanka document high levels of provider prejudice toward sex workers, PWID, and transgender individuals, leading to delayed presentation and poor retention (13, 119, 120). Training programs reduce discriminatory attitudes by up to 60% when combined with institutional accountability measures, yet most countries lack mandatory, standardized non-discrimination curricula for health cadres (84, 121). Structural stigma also manifests in service location and design—centralized hospital-based ART services in Sri Lanka and Pakistan exclude rural and mobile populations, while harm reduction programs in India are subject to police raids, deterring PWID from accessing needle-syringe exchange and OST (59, 62, 122, 123). Health system transformation must prioritize culture change through leadership engagement, inclusive policies, and routine monitoring of stigma indicators alongside clinical outcomes (124, 125).

### 5.4 Community participation as a catalyst

Civil society organizations (CSOs) have been the linchpin of effective HIV responses, especially in reaching marginalized groups. Peer-led outreach by the Durbar Mahila Samanwaya Committee in India and Blue Diamond Society in Nepal has achieved high testing and retention rates among female sex workers and transgender persons, respectively (15, 61, 126). However, CSOs often operate under restrictive legal frameworks and with inadequate funding; Pakistan allocates only 5% of its HIV budget to community services, undermining scale-up potential (16, 66). Evidence indicates that every 10% increase in community-led service funding correlates with a 5% improvement in cascade outcomes among key populations (127). Institutionalizing community participation requires formal governance mechanisms—reserved seats on national AIDS boards, budgetary earmarks, and co-creation of program strategies—to ensure that affected populations shape the services designed for them (69, 107).

### 5.5 Comparative and global implications

South Asia’s experience underscores that legal change is necessary but not sufficient; it must be accompanied by robust implementation, stigma reduction, service redesign, and community empowerment to realize full public health benefits. The causal pathway from rights-based governance to epidemic control is evidenced by regional correlations between RBA score (aggregated dimension ratings) and 95-95-95 achievements: higher RBA scores align with stronger cascade performance (128, 129). This framework offers a model for other regions, such as Eastern Europe and Central Asia, where punitive laws and service gaps mirror South Asian challenges (130, 131).

### 5.6 Policy recommendations revisited

The four strategic pillars—legal reform, health system transformation, funding equity, and regional collaboration—offer synergistic opportunities. Modeling from decriminalization in Portugal and harm reduction in Ukraine suggests that integrating these pillars can reduce HIV incidence by 30%–45% over a decade (132, 133). Policymakers should prioritize intersecting interventions, such as combining decriminalization pilots with community-led service expansion and anti-stigma training, to maximize impact and generate politically salient successes early in the reform process (134).

### 5.7 Strengths and limitations

This study’s strength lies in its comprehensive, comparative approach, integrating legal analysis, policy review, program data, and community perspectives across four diverse national contexts. Employing a structured RBA framework ensures consistency and facilitates actionable insights. Limitations include reliance on secondary sources, variable data quality across countries, and limited disaggregation of outcome data for key populations. Future research should incorporate primary qualitative studies with affected individuals and service providers to deepen understanding of implementation barriers and facilitators (135, 136).

### 5.8 Balancing legal mandates and public health ethics

While legal reforms such as decriminalization, anti-discrimination statutes, and constitutional protections are critical milestones in advancing the rights of people living with HIV (PLHIV), they cannot by themselves guarantee meaningful change. Evidence from national and regional contexts demonstrates that statutory protections often coexist with coercive practices such as mandatory HIV testing, travel restrictions, and punitive disclosure requirements. These measures, although frequently justified in the name of protecting public health, can in practice undermine autonomy, confidentiality, and equitable access to care, thereby reinforcing stigma rather than alleviating it (137, 138).

A rights-based approach therefore requires more than the enactment of laws; it demands the integration of public health ethics into policy design and implementation. Ethical principles of justice, non-maleficence, autonomy, and solidarity provide the normative foundation to ensure that legal reforms advance health equity. For example, laws that prohibit discrimination must be accompanied by provider training, community engagement, and mechanisms of accountability to prevent violations in healthcare settings. Similarly, protections for key populations must be enforced through supportive rather than punitive health systems that build trust with marginalized communities.

By aligning legal frameworks with ethical imperatives, governments can move beyond symbolic compliance toward reforms that tangibly reduce stigma, expand access, and improve health outcomes. This balance between the structural guarantees of law and the lived realities of ethical practice is central to achieving sustainable progress in HIV prevention and care.

## 6 Conclusion

This comparative rights-based policy review demonstrates that legal and structural reforms are fundamental to HIV epidemic control in South Asia. Countries that have implemented progressive decriminalization—such as India’s repeal of Section 377 and Nepal’s constitutional recognition of third gender status—show substantial improvements in HIV diagnosis, treatment uptake, and viral suppression among key populations. Conversely, Pakistan and Sri Lanka’s retention of punitive laws correlates with persistent service gaps and rising incidence among MSM, PWID, and transgender individuals (14, 15).

Achieving the UNAIDS 95-95-95 targets by 2025 and beyond requires sustained action across four strategic pillars: (1) legal reform and decriminalization to remove punitive barriers and enable harm reduction, (2) health system transformation to decentralize services, scale differentiated delivery, and institutionalize non-discrimination, (3) funding equity and community empowerment to ensure at least 30% of HIV resources reach community-led programs, and (4) regional collaboration to harmonize policies and share best practices across borders. Modelling from diverse jurisdictions suggests that integrated implementation of these pillars can reduce HIV incidence by up to 45% over a decade and accelerate progress toward universal viral suppression.

Structural stigma within healthcare settings remains a critical barrier; provider training and accountability measures must be prioritized to create truly inclusive services for all populations, irrespective of legal status or social identity. Legal protections without enforcement mechanisms offer limited benefit and may erode community trust if unaccompanied by institutional capacity-building for rights commissions and judicial bodies (8).

Civil society organizations are indispensable in reaching hidden and criminalized groups, yet operate under restrictive legal and funding constraints. Formalizing community participation within national HIV governance structures—through reserved seats, budget earmarks, and decision-making authority—will leverage their unique capacity for peer-led outreach and advocacy.

The implementation gap between law reform and practice underscores the need for continuous monitoring and evaluation of legal environment indicators alongside epidemiological outcomes. National human rights institutions and parliamentary committees should conduct regular reviews of discriminatory laws, service access inequities, and enforcement practices, ensuring real-world accountability for legal reforms.

In conclusion, sustainable HIV epidemic control in South Asia demands a rights-based governance paradigm that integrates legal reform, service innovation, community leadership, and regional cooperation. By aligning policy with human rights principles and empirical evidence, South Asian nations can not only achieve the 95-95-95 goals but also advance social justice, equity, and health for all. Failure to act on these interconnected strategies risks perpetuating cycles of criminalization, stigma, and poor health outcomes—undermining both public health imperatives and fundamental human rights.

This comparative analysis demonstrates that rights-based reforms are essential for moving from a punitive, criminalization-centered model of HIV governance toward one rooted in care, dignity, and equity. However, legal mandates alone cannot guarantee transformative change. As evidence from Iran and across Asia and the Middle East illustrates, statutory protections often remain under-implemented or coexist with coercive measures such as mandatory testing and travel restrictions, which undermine the very rights they claim to protect.

To be effective, rights-based reforms must be accompanied by an explicit commitment to public health ethics. Principles of justice, autonomy, non-maleficence, and solidarity provide the ethical grounding necessary to ensure that legal reforms reduce stigma, minimize discrimination, and promote equitable access to services. By aligning legal frameworks with ethical practice—through confidentiality protections, non-discriminatory service delivery, and community-based interventions—governments can ensure that reforms are not merely symbolic but deliver tangible improvements in the lives of people living with HIV.

Ultimately, achieving the transition from criminalization to care requires striking a balance between the structural guarantees provided by law and the lived realities shaped by ethical practice. Only when legal mandates and public health ethics converge can HIV responses advance health equity and human rights in meaningful and sustainable ways.

## 7 Limitations

As this work is based on a narrative review, certain methodological constraints should be acknowledged. The synthesis may be influenced by interpretive or confirmation bias, and findings are dependent on the availability and quality of publicly accessible documents. Although triangulation across multiple data sources was applied to enhance reliability, the absence of quantitative synthesis and formal inclusion–exclusion procedures limits reproducibility. Nevertheless, this approach provides valuable conceptual depth and theoretical insight into how legal and policy reforms intersect with public health ethics and human rights in the region.

## References

[1] UNAIDS. Global AIDS Update 2024. Geneva: UNAIDS (2024).

[2] WHO. HIV/AIDS in the South-East Asia region: Progress and challenges. New Delhi: WHO SEARO (2023). 10.1016/S2055-6640(20)31092-X PMC5353351 28303199

[3] UNAIDS. UNAIDS Data 2024. Geneva: UNAIDS (2024).

[4] Beyrer C Baral SD Griensven F Goodreau SM Chariyalertsak S Wirtz AL. Global epidemiology of HIV infection in men who have sex with men. Lancet. (2012) 380:367–77. doi: 10.1016/S0140-6736(12)60821-6 22819660 PMC3805037

[5] UNDP UNAIDS. HIV and the law: Risks, Rights & Health (2012): Global commission report. New York: UNDP (2012).

[6] UNAIDS. Criminalization and HIV: Policy brief and evidence summaries. Geneva: UNAIDS (2024).

[7] Shannon K Strathdee SA Goldenberg SM Duff P Mwangi P Rusakaniko S. Global epidemiology of HIV among female sex workers: influence of structural determinants. Lancet. (2015) 385:55–71. doi: 10.1016/S0140-6736(14)60931-4 25059947 PMC4297548

[8] Government of India. The HIV and AIDS (prevention and control) act. New Delhi: Ministry of Law & Justice (2017).

[9] UNAIDS. Human rights-based approach to ending AIDS. Geneva: UNAIDS (2022).

[10] UNAIDS. Rights-based monitoring and evaluation of national HIV responses. Geneva: UNAIDS (2021).

[11] UNAIDS. Communities at the Centre: Defending rights, breaking barriers, reaching people with HIV services. Geneva: UNAIDS (2020).

[12] Office of the United Nations High Commissioner for Human Rights. HIV/AIDS and human rights—international guidelines. Geneva: OHCHR (2006).

[13] Ramesh BM Ganju D Mahapatra B Mishra RM Saggurti N. Changes in risk behaviours and prevalence of sexually transmitted infections following HIV preventive interventions among female sex workers in five districts in Karnataka state, south India. Sexually Transm Infec. 86:i17–24. doi: 10.1136/sti.2009.038513 PMC3252604 20167725

[14] v NSJ. Union of India. (2018). Available online at: https://main.sci.gov.in

[15] Pant SB, v O. Nepal Government. Supreme Court of Nepal; (2007). Available online at: https://www.refworld.org/cases

[16] National AIDS Control Organisation (NACO). India HIV Estimates 2023. New Delhi: NACO (2023).

[17] Government of Pakistan. Transgender persons (protection of rights) act, 2018. Islamabad: Government of Pakistan (2018).

[18] National STD/AIDS Control Programme. Sri Lanka country profile 2024. Colombo: Ministry of Health (2024).

[19] UNAIDS. UNAIDS Data 2023. Geneva: UNAIDS (2023).

[20] WHO. Global health sector strategies on HIV, viral hepatitis and sexually transmitted infections 2022–2030. Geneva: WHO (2022).

[21] Beyrer C Wirtz AL Walker D Johns B Sifakis F Baral S. The global HIV epidemics among men who have sex with men. World Bank Publications - Books. https://ideas.repec.org//b/wbk/wbpubs/2308.html

[22] Gruskin S Tarantola D. HIV/AIDS and human rights revisited. Canadian HIV/AIDS Policy Law Rev. (2001) 6:24–9. Available online at: https://pubmed.ncbi.nlm.nih.gov/11837018/11837018

[23] Logie CH Turan JM. How do we balance tensions between COVID-19 public health responses and stigma mitigation? Learning from HIV research. *AIDS Behav*. (2020) 24:2003–6. doi: 10.1007/s10461-020-02856-8 32266502 PMC7137404

[24] UNAIDS. Fast-track: Ending the AIDS epidemic by 2030. Geneva: UNAIDS (2014).

[25] UNAIDS. India country fact sheet 2023. Geneva: UNAIDS (2023).

[26] UNAIDS. Pakistan country fact sheet 2023. Geneva: UNAIDS (2023).

[27] Ministry of Health Sri Lanka. National Strategic Plan on HIV and AIDS 2023–2027. Colombo: Ministry of Health (2023).

[28] Mude W Mwenyango H Preston R O’Mullan C Vaughan G Jones G. HIV Testing Disruptions and Service Adaptations During the COVID-19 Pandemic: A Systematic Literature Review. AIDS Behav. (2024) 28:186–200. doi: 10.1007/s10461-023-04139-4 37548796 PMC10803448

[29] UNAIDS. Putting people at the Centre of HIV and AIDS responses. Geneva: UNAIDS (2014).

[30] WHO. Consolidated guidelines on HIV prevention, testing, treatment, service delivery and monitoring: Recommendations for a public health approach. Geneva: WHO (2021). 34370423

[31] Gruskin S Mills EJ Tarantola D. History, principles, and practice of health and human rights. Lancet. (2007) 370:449–55. doi: 10.1016/S0140-6736(07)61200-8 17679022

[32] WHO. Consolidated guidelines on HIV prevention, diagnosis, treatment and care for key populations. Geneva: WHO (2016). 27559558

[33] OHCHR. Fact sheet: HIV and human rights. Geneva: OHCHR (2024).

[34] UNAIDS. Rights-based approach to ending AIDS as a public health threat. Geneva: UNAIDS (2022).

[35] UNAIDS. Rights-based monitoring and evaluation: Practical guidance. Geneva: UNAIDS (2021).

[36] UNAIDS. Global AIDS monitoring 2024: Indicators and guidance. Geneva: UNAIDS (2024).

[37] Arthur M Saha R Kapilashrami A. Community participation and stakeholder engagement in determining health service coverage: A systematic review and framework synthesis to assess effectiveness. J Global Health. (2023) 13:04034. doi: 10.7189/jogh.13.04034 PMC10173679 37166063

[38] WHO. Service delivery approaches to HIV prevention, testing, care and treatment. Geneva: WHO (2019).

[39] UNAIDS. Country operational guidance for key populations. Geneva: UNAIDS (2016).

[40] WHO. Global health sector strategy on HIV 2022–2030. Geneva: WHO (2022).

[41] Global Fund to Fight AIDS. Community M. Rights and gender strategic initiative. Geneva: Global Fund (2023).

[42] Global Fund. Communities delegation: Empowering communities for impact. Geneva: Global Fund (2017).

[43] UNAIDS. Communities at the Centre: People living with HIV as co-creators of responses. Geneva: UNAIDS (2017).

[44] UNAIDS. Global AIDS monitoring 2023: Guidance and indicators. Geneva: UNAIDS (2023).

[45] WHO. Policy brief: Policy audit tool for HIV, health and human rights. Geneva: WHO (2019).

[46] Lauer JA Sassi F Soucat A Vigo A. Health Taxes: Policy and Practice. World Scientific Publishing Company. (2023). doi: 10.1142/q0365

[47] UNAIDS. Police and HIV: Good practice for reducing risk among sex workers. Geneva: UNAIDS (2014).

[48] Decker MR Crago A-L Chu SKH Sherman SG Seshu MS Buthelezi K . Human rights violations against sex workers: Burden and effect on HIV. Lancet. (2015) 385:186–99. doi: 10.1016/S0140-6736(14)60800-X 25059943 PMC4454473

[49] Beyrer C Baral S Collins C Richardson ET Sullivan PS Sanchez J. The global response to HIV in men who have sex with men. Lancet. (2016) 388:198–206. doi: 10.1016/S0140-6736(16)30781-4 27411880

[50] NACP. Pakistan HIV response progress update 2023. Islamabad: Ministry of National Health Services (2023).

[51] Rao S Gupta R Mishra S. Impact of decriminalization on HIV testing among MSM in India: quasi-experimental evidence. BMC Public Health. (2020):20. doi: 10.1186/s12889-020-09322-331910835

[52] UNAIDS. The dangerous inequalities report. Geneva: UNAIDS (2022).

[53] Stringer KL Turan B McCormick L Durojaiye M Nyblade L Kempf M-C . HIV-Related Stigma among Healthcare Providers in the Deep South. AIDS Behav. (2016) 20:115–25. doi: 10.1007/s10461-015-1256-y 26650383 PMC4718797

[54] Kaur J Singh R. Awareness and implementation of India’s HIV/AIDS act among healthcare workers in Punjab. J Clin Diagn Res. (2019):13. Available online at: https://www.jcdr.net

[55] NCASC. National HIV estimates 2023. Kathmandu: Ministry of Health and Population (2023).

[56] Giri SK. The Rule of Law Under the Constitution of Nepal. Gyanjyoti. (2023) 3:71–80. doi: 10.3126/gyanjyoti.v3i1.53038

[57] NSACP. Annual report 2023. Colombo: Ministry of Health (2024).

[58] WHO. Progress on pre-exposure prophylaxis (PrEP) implementation in Asia and the Pacific—2024 update. Manila: WHO WPRO/SEARO (2024).

[59] Goswami S Borate S Marupuru S Marupuru S. A Scoping Review of the Current Landscape of Pre-Exposure Prophylaxis and Postexposure Prophylaxis in India. AIDS Patient Care and STDs. (2024) 38:287–304. doi: 10.1089/apc.2024.0078 38800957

[60] AVAC. PrEPWatch: Global PrEP tracker. New York: AVAC (2024).

[61] The Global Fund. Modular Framework Handbook—HIV. Geneva: The Global Fund (2023).

[62] WHO. Implementation tool for pre-exposure prophylaxis (PrEP) of HIV infection. Geneva: WHO (2019).

[63] WHO. Consolidated guidelines on HIV, viral hepatitis and STI prevention, diagnosis, treatment and care for key populations. Geneva: WHO (2022). 36417550

[64] Bulstra CA Hontelez JAC Otto M Stepanova A Lamontagne E Yakusik A . Integrating HIV services and other health services: A systematic review and meta-analysis. PLoS Med. (2021) 18:e1003836. doi: 10.1371/journal.pmed.1003836 34752477 PMC8577772

[65] UNAIDS. Understanding measures of progress towards the 95–95–95 HIV testing, treatment and viral suppression targets. Geneva: UNAIDS.

[66] Global Commission on Drug Policy. Advancing health and human rights: Decriminalization of drug use. Geneva: GCDP (2019).

[67] WHO. Technical guide for countries to set targets for universal access to HIV prevention, treatment and care for injecting drug users. Geneva: WHO/UNAIDS/UNODC (2012).

[68] Naz Foundation (India) Trust. Community-led HIV intervention impact report 2022–23. New Delhi: Naz (2023).

[69] WHO. Community engagement in health decision-making: Evidence and guidance. Geneva: WHO (2021).

[70] UNAIDS. Human rights-based approach: Operational guidance for national HIV responses. Geneva: UNAIDS (2022).

[71] WHO. HIV, health and human rights policy audit tool. Geneva: WHO (2019).

[72] UNAIDS. Police in action: Reducing HIV risk among sex workers. Geneva: UNAIDS (2014).

[73] Hughes CE Stevens A. What can we learn from the Portuguese decriminalization of illicit drugs? Br J Criminol. (2010) 50:999–1022. doi: 10.1093/bjc/azq038

[74] INPUD. Good practice guide for policing and harm reduction. London: International Network of People who Use Drugs (2018).

[75] HRW. “We have the right to live”: Transgender rights and state response in Pakistan. New York: HRW (2020).

[76] UNDP. Legal recognition of gender identity: Global standards and best practices. New York: UNDP (2019).

[77] Karan A Negandhi H Hussain S Zapata T Mairembam D Graeve H. Size, composition and distribution of health workforce in India: why, and where to invest? . (2021) 19. doi: 10.1186/s12960-021-00572-2 PMC7983088 33752675

[78] Secretariat SAARC. Regional framework for HIV and human rights. Kathmandu: SAARC (2022).

[79] UNDP. Strengthening national human rights institutions for HIV responses. New York: UNDP (2021).

[80] Inter-Parliamentary Union UNAIDS. Handbook for parliamentarians on HIV and the law. Geneva: IPU/UNAIDS (2020).

[81] Niyigena A Girukubonye I Barnhart DA Cubaka VK Niyigena PC Nshunguyabahizi M . Rwanda’s community health workers at the front line: A mixed-method study on perceived needs and challenges for community-based healthcare delivery during COVID-19 pandemic. BMJ Open. (2022) 12:e055119. doi: 10.1136/bmjopen-2021-055119 PMC9058292 35487742

[82] WHO. WHO guideline on community health workers (update [internet]). Geneva: WHO (2022).

[83] Frontières MS. Reaching the last mile: Mobile HIV services in fragile settings. Geneva: MSF (2018).

[84] UNDP. Training manual on HIV, human rights and stigma reduction for health workers. New York: UNDP (2020).

[85] UNAIDS. Health worker sensitivity training toolkit. Geneva: UNAIDS (2019).

[86] WHO. Differentiated service delivery for HIV treatment: Policy brief. Geneva: WHO (2021).

[87] Grimsrud A Bygrave H Doherty M. Reimagining HIV service delivery: the role of differentiated models. J Int AIDS Soc. (2016):19. doi: 10.7448/IAS.19.1.21484 PMC5136137 27914186

[88] AVAC. PrEPWatch: Country updates and dashboards. New York: AVAC (2024).

[89] UNAIDS. Innovative financing for HIV responses. Geneva: UNAIDS (2021).

[90] International Drug Policy Consortium. Hepatitis C and HIV harm reduction: A practical handbook. London: IDPC (2019).

[91] Sudjaritruk T Aurpibul L Songtaweesin WN Narkpongphun A Thisayakorn P Chotecharoentanan T . Integration of mental health services into HIV healthcare facilities among Thai adolescents and young adults living with HIV. J Intern AIDS Soc. (2021) 24:e25668. doi: 10.1002/jia2.25668 PMC7876472 33569878

[92] WHO. Guidance on integrating mental health into HIV services. Geneva: WHO (2024).

[93] World Bank. Universal health coverage for inclusive and sustainable growth: HIV financing perspectives. Washington, DC: World Bank (2022).

[94] WHO. Financial protection and HIV: Monitoring out-of-pocket costs and catastrophic expenditure. Geneva: WHO (2019).

[95] PEPFAR/USAID. Quality assurance and improvement for HIV services: Implementation guide. Washington, DC: PEPFAR (2021).

[96] WHO. Standards for improving quality of HIV care. Geneva: WHO (2018).

[97] World Bank. Performance-based financing in health: Lessons for HIV programs. Washington, DC: World Bank (2016).

[98] UNAIDS. Financing the HIV response: The case for community-led services. Geneva: UNAIDS (2023).

[99] WHO. Guideline on community engagement for quality, people-centred and resilient health services. Geneva: WHO (2022).

[100] The Global Fund. Community-led monitoring: Guidelines and tools. Geneva: The Global Fund (2019).

[101] The Global Fund. Community, rights and gender technical assistance program—Guidance note. Geneva: The Global Fund (2020).

[102] Ministry of Home Affairs (India). Foreign contribution (regulation) amendment act, 2020. New Delhi: Government of India (2020).

[103] World Bank. Innovative financing instruments for health: a review. Washington, DC: World Bank (2018).

[104] CIVICUS. Multi-year funding for civil society: Why it matters. Johannesburg: CIVICUS (2021).

[105] UNAIDS. Establishing multi-stakeholder HIV coordination mechanisms: Guidance for inclusion and accountability. Geneva: UNAIDS (2020).

[106] UNAIDS. Principles of meaningful engagement of people living with HIV and key populations. Geneva: UNAIDS (2021).

[107] The Global Fund. Community systems strengthening module. Geneva: The Global Fund (2022).

[108] UNAIDS. Sustainable financing for community-led HIV responses. Geneva: UNAIDS (2021).

[109] CIVICUS. Social enterprise models for sustainability in civil society. Johannesburg: CIVICUS (2019).

[110] Beyrer C Baral S Collins C Richardson ET Sullivan PS Sanchez J. The high-risk context of criminalization and HIV outcomes: a systematic review. Lancet HIV. (2018):5. doi: 10.1016/S2352-3018(18)30130-1

[111] Jesseman R. (n.d.). Decriminalization: Options and Evidence. Available online at: https://shorturl.at/LVBdw

[112] Dowdy DW Powers KA Hallett TB. Towards evidence-based integration of services for HIV, non-communicable diseases and substance use: Insights from modelling. J Intern AIDS Soc. (2020) 23:e25525. doi: 10.1002/jia2.25525 PMC7305415 32562385

[113] Viswasam N Rivera J Comins C Rao A Lyons CE Baral S. The Epidemiology of HIV Among Sex Workers Around the World: Implications for Research, Programmes, and Policy In: Goldenberg SM Morgan Thomas R Forbes A Baral S, editors. Sex Work, Health, and Human Rights: Global Inequities, Challenges, and Opportunities for Action: Springer (2021). Available online at: http://www.ncbi.nlm.nih.gov/books/NBK585689/36315785

[114] Suthar AB Bui DD Granich RM Nadol P Tran HV Sabin K . The potential impact of expanding antiretroviral therapy and combination prevention in Vietnam: The modeling approach. J Acquir Immune Defici Syndromes. (2014) 1999:e62–3. doi: 10.1097/QAI.0000000000000139 24828271

[115] Gupta S Granich R. When will sub-Saharan Africa adopt HIV treatment for all? South Afr. J HIV Med. (2016):17. doi: 10.4102/sajhivmed.v17i1.459 PMC5843218 29568615

[116] Braveman P Gruskin S. Defining equity in health. J Epidemiol Community Health. (2003) 57:254–8. doi: 10.1136/jech.57.4.254 12646539 PMC1732430

[117] Nampewo Z Mike JH Wolff J. Respecting, protecting and fulfilling the human right to health. Intern J Equity Health. (2022) 21:36. doi: 10.1186/s12939-022-01634-3 PMC8922072 35292027

[118] Dessie ZG Zewotir T. HIV-related stigma and associated factors: A systematic review and meta-analysis. Front Public Health. (2024) 12. doi: 10.3389/fpubh.2024.1356430 PMC11300231 39109161

[119] Sison OT Baja ES Bermudez ANC Quilantang M N I Dalmacion GV . Association of anticipated HIV testing stigma and provider mistrust on preference for HIV self-testing among cisgender men who have sex with men in the Philippines. BMC Public Health. (2022) 22:2362. doi: 10.1186/s12889-022-14834-x 36527003 PMC9756449

[120] Sukhera J Knaak S. A realist review of interventions to dismantle mental health and substance use related structural stigma in healthcare settings. SSM - Mental Health. (2022) 2:100170. doi: 10.1016/j.ssmmh.2022.100170

[121] SEARO WHO. HIV drug resistance report: South-East Asia. New Delhi: WHO SEARO (2023).

[122] The Global Fund. Pakistan harm reduction program review 2022. Geneva: The Global Fund (2022).

[123] Nyblade L Reddy A Mbote D. Implementing health facility interventions to reduce HIV stigma. J Int AIDS Soc. (2019):22. doi: 10.1002/jia2.25325

[124] Hatzenbuehler ML Link BG. Introduction to structural stigma and health. Soc Sci Med. (2014) 103:1–6. doi: 10.1016/j.socscimed.2013.12.017 24445152

[125] Patel SK Prabhakar P Jain AK Saggurti N Adhikary R. Relationship between Community Collectivization and Financial Vulnerability of Female Sex Workers in Southern India. Plos One. (2016) 11:e0156060. doi: 10.1371/journal.pone.0156060 27227998 PMC4881938

[126] UNAIDS. The case for investing in community-led HIV responses. Geneva: UNAIDS (2022).

[127] Implementing_Comprehensive_HIV_and_HCV_Programmes_with_People_Who_Inject_Drugs_PRACTICAL_GUIDANCE_FOR_COLLABORATIVE_INTERVENTIONS. Available online at: https://www.unodc.org/documents/hiv-aids/publications/Implementing_Comprehensive_HIV_and_HCV_Programmes_with_People_Who_Inject_Drugs_PRACTICAL_GUIDANCE_FOR_COLLABORATIVE_INTERVENTIONS.pdf

[128] Eisinger RW Dieffenbach CW Fauci AS. HIV viral load and transmissibility of HIV infection: undetectable equals untransmittable. JAMA. (2019) 321:451–2. doi: 10.1001/jama.2018.21167 30629090

[129] DeBeck K Cheng T Montaner JS Beyrer C Elliott R Sherman S . HIV and the Criminalization of Drug Use Among People who Inject Drugs: A Systematic Review. The Lancet. HIV. (2017) 4:e357–74. doi: 10.1016/S2352-3018(17)30073-5 28515014 PMC6005363

[130] Mumtaz GR Awad SF Feizzadeh A Weiss HA Abu-Raddad LJ. HIV incidence among people who inject drugs in the Middle East and North Africa: Mathematical modelling analysis. J Intern AIDS Soc. (2018) 21:e25102. doi: 10.1002/jia2.25102 PMC5867334 29577623

[131] Kremer C Kamali A Kuteesa M Seeley J Hens N Nsubuga RN. Modelling the impact of combining HIV prevention interventions on HIV dynamics in fishing communities in Uganda. BMC Infectious Diseases. (2023) 23:173. doi: 10.1186/s12879-023-08113-2 36949387 PMC10031877

[132] Hughes CE Stevens A. What Can We Learn From The Portuguese Decriminalization of Illicit Drugs. British J Crimin. (2010) 50:999–1022. doi: 10.1093/bjc/azq038

[133] Brief Technical: Harm Reduction for People who Use Drugs. (n.d.). Available online at: https://www.aidsdatahub.org/sites/default/files/resource/global-fund-harm-reduction-briefing-2020.pdf

[134] Campbell C Scott K Nhamo M. Social capital and community responses to HIV: qualitative insights. AIDS Care. (2013) 25. doi: 10.1080/09540121.2012.748170 PMC3701935 23745625

[135] Nyblade L Mingkwan P Stockton MA. Stigma reduction: An essential ingredient to ending AIDS by 2030. Lancet HIV. (2021) 8:e106–13. doi: 10.1016/S2352-3018(20)30309-X 33539757

[136] Akrami F Gouya MM Doroudi F. Ethical evaluation of strategic plan for HIV prevention and control: steps to minimize discrimination and health equity (a case study in Iran). Ethics Med Public Health. (2022) 23:100798. doi: 10.1016/j.jemep.2022.100798

[137] Akrami F Dauda B. Perspectives on HIV stigma in Asia and Middle East: legal protections, ethical considerations, and public health solutions. J Res Develop Nursing Midwifery. 21:29–30. doi: 10.61882/jgbfnm.21.4.29

[138] WHO. Regional cross-border health cooperation toolkit. Geneva: WHO (2018).

[139] PEPFAR. Monitoring and evaluation guidance for stigma and discrimination reduction. Washington, DC: PEPFAR (2020).

[140] NACO. National Strategic Plan for HIV/AIDS and STI 2023–2030. New Delhi: Ministry of Health & Family Welfare (2023).

